# Summer effects on body mass index (BMI) gain and growth patterns of American Indian children from kindergarten to first grade: a prospective study

**DOI:** 10.1186/1471-2458-11-951

**Published:** 2011-12-23

**Authors:** Jianduan Zhang, John H Himes, Peter J Hannan, Chrisa Arcan, Mary Smyth, Bonnie Holy Rock, Mary Story

**Affiliations:** 1 Division of Epidemiology and Community Health, School of, Public Health, University of Minnesota, 1300 South 2nd Street, Suite 300, Minneapolis, MN 55454, USA; 2 Department of Maternal and Children Care and Adolescent Health, School of Public Health, Huazhong University of Science and Technology, Wuhan, Hubei, China 430030

**Keywords:** American Indian, children, body mass index, summer vacation

## Abstract

**Background:**

Overweight and obesity are highly prevalent among American Indian children, especially those living on reservations. There is little scientific evidence about the effects of summer vacation on obesity development in children. The purpose of this study was to investigate the effects of summer vacation between kindergarten and first grade on growth in height, weight, and body mass index (BMI) for a sample of American Indian children.

**Methods:**

Children had their height and weight measured in four rounds of data collection (yielded three intervals: kindergarten, summer vacation, and first grade) as part of a school-based obesity prevention trial (Bright Start) in a Northern Plains Indian Reservation. Demographic variables were collected at baseline from parent surveys. Growth velocities (Z-score units/year) for BMI, weight, and height were estimated and compared for each interval using generalized linear mixed models.

**Results:**

The children were taller and heavier than median of same age counterparts. Height Z-scores were positively associated with increasing weight status category. The mean weight velocity during summer was significantly less than during the school year. More rapid growth velocity in height during summer than during school year was observed. Obese children gained less adjusted-BMI in the first grade after gaining more than their counterparts during the previous two intervals. No statistically significant interval effects were found for height and BMI velocities.

**Conclusions:**

There was no indication of a significant summer effect on children's BMI. Rather than seasonal or school-related patterns, the predominant pattern indicated by weight-Z and BMI-Z velocities might be related to age or maturation.

**Trial registration:**

Bright Start: Obesity Prevention in American Indian Children Clinical Trial Govt ID# NCT00123032

## Background

The dramatic improvement in the nutritional status of the American Indian populations since the late 1960s has been accompanied by the emergence of obesity as a serious public health problem [1]. A secular trend of increasing overweight among Navajo Indian children 6-12 years of age has been documented [2]. The study indicated that American Indian children aged 6-17 years from rural school districts on or near reservations bear the burden of excess weight relative to other racial and ethnic groups, with a combined prevalence of overweight and obesity up to 53.8% [3], 2-3 times higher than the national average [4,5].

The concurrent and long-term adverse consequences of obesity in childhood are well documented, including asthma, Type 2 diabetes, and elevated cardiovascular disease risks, including hypercholesterolemia and hypertension [6,7]. Type 2 diabetes, long considered an adult illness, is becoming more common among American Indian children aged 10 years and older [8]. A recent study concluded that obesity in childhood is a primary factor contributing to increased rates of premature death from endogenous causes in American Indian populations [9].

While obesity occurs as the result of interactions between genetic and environmental factors [10], school and away-from-school circumstances related to food availability and quality and to levels of physical activity are factors that may contribute to childhood obesity risk in school-age children. Studies also emphasize the influence of non-school environmental factors, such as low socio-economic status [11], high consumption of fast food and energy-dense convenience foods [12], lack of facilities and opportunities for physical activity, and lack of parental supervision among the general population [13].

Schools have become important venues for interventions to prevent childhood obesity [14-16]. However, a concern for those developing and implementing school-based interventions has been the effect of the summer months when children no longer participate in school-based activities and are exposed to factors that may contribute to excess weight gain. From an intervention perspective, it is important to understand if the less structured circumstances during summer vacation weaken or even negate any benefits gained by the intervention during the school year.

There is little scientific evidence about the effects of summer vacation on obesity development in children, and the few existing studies yield inconsistent results. While one study indicated that body mass index (BMI) increments during out-of-school periods are likely due to normal growth among American Indian children in grades 3-8 (9-14 years) [17], others showed that children are more likely to gain BMI during the summer than during the school year [18], and that improvements of a school-year obesity intervention might be lost during summer vacation [19].

The time from kindergarten to first grade (approximately 5-7 years of age) is a critical transition period for the development of body mass index, the most common and recommended measure of overweight and obesity in children [20]. We investigated the effects of summer vacation between kindergarten and first grade on growth in height, weight, and BMI for a sample of American Indian Children participating in a school-based intervention program targeting prevention of excessive weight gain. Our focus was to investigate the impact of summer away from school irrespective of the intervention, in order to use the full sample and produce more generalizable results.

## Methods

A total of 454 American Indian children aged 4.8-7.9 years old attending kindergarten at 14 schools located on the Pine Ridge Reservation (including one school just outside the border) in South Dakota, were enrolled into a cluster-randomized, controlled trial (Bright Start). The Bright Start study followed the children from the beginning of kindergarten until the completion of first grade. The study was designed to develop and test the efficacy of a school environmental intervention, augmented with a family household environmental intervention, to reduce excessive weight gain by increasing physical activity and healthy dietary practices. The focus of Bright Start was to create dietary and physical activity environmental change at school and home. The intervention had three primary goals: 1) increase physical activity at school to a minimum of 60 min/day via recess, classroom walking program, classroom action breaks, and physical education class; 2) modify school meals and snacks provided by the school food service; and 3) involve parents/caregivers in making behavioural and environmental changes at home by way of family night events that included interactive informational booths, hands-on demonstrations, and family goal-setting with follow-up motivational calls to those parents/caregivers who set goals. The 14 schools were randomly assigned to intervention and control conditions after baseline measurements. Two successive cohorts of schools, each including both conditions were followed for two school years.

Baseline anthropometric data for the two cohorts were collected in fall 2005 and 2006, respectively, near the beginning of the school years. For each cohort, round 2 data were collected the following spring, near the end of the school year (mean interval, 144.3 days; SD, 22.5 days), round 3 data the subsequent fall (mean interval 167.6 days; SD, 20.7 days), and round 4 data the following spring (mean interval, 226.4 days; SD, 22.2 days). These four rounds of data collection yielded three intervals corresponding to kindergarten, summer vacation, and first grade, respectively. Data collection for round 2 was conducted before school was dismissed in the spring, and round 3 was collected after school started in the fall, so the actual interval between measurement occasions corresponding to summer vacation was somewhat longer than actual time out of school. Also, there was some variation across schools in the actual dates between data collection in rounds 2 and 3. Consequently, to standardize and estimate Z-score velocities, the interval for summer vacation was adjusted linearly to the period between May18th through August 18th, the approximate average interval across all schools for actual summer vacation.

Body weight (kg) was measured to the nearest 100 g using a Tanita (model 300) scale, with subjects wearing light indoor clothing. Height (cm) was measured using a portable stadiometer (Perspective Enterprises, Portage, MI) to the nearest 0.1 cm. Data collectors were centrally trained and used standardized protocols recommended by Lohman et al. [21]. Body mass index (BMI) was calculated as weight (kg)/height (m)^2^. The prevalences of overweight and obesity were estimated relative to the Centers for Disease Control (CDC) Growth Charts 2000 [22]. Children with BMI < 85th percentile of the age- and gender-specific reference were considered to be at normal weight; BMI ≥ 85th but < 95th percentiles was considered as "overweight," and BMI ≥ 95th percentile was identified as "obese" [20]. There were no meaningful differences by gender groups so the data were pooled for these analyses, although gender was always included in the adjusted models. The final number of children with complete data was 440. Demographic variables were collected at baseline from parent surveys, including gender, birth date of children, parental and household characteristics, etc. Parents signed written informed consent giving permission for their children and themselves to participate in all Bright Start measurements.

The administrators and boards from participating schools and districts approved and supported participation in the study. Ethical and human subject approvals were obtained from the Institutional Review Boards (IRB) of the University of Minnesota, the Oglala Sioux Tribal Council, and the Aberdeen Area Indian Health Service.

To estimate the mean Z-scores at each interval, growth trajectories (time trend) of BMI, weight and height Z-scores were calculated and adjusted linearly to a 20-month interval from baseline to round 4, with exactly 8 months in kindergarten, followed by 4 months in summer vacation, and 8 months in first grade. For example, adjusted BMI Z _round 2 _= BMI Z _baseline _+ BMI Z velocity (Z/y) _kindergarten _× 8/12; and adjusted BMI Z _round 3 _= adjusted BMI Z _round 2 _+ BMI Z velocity (Z/y) _summer _× 4/12, etc. BMI Z-scores adjusted to the exact intervals were back-transformed to the natural units (kg/m^2^) using the published LMS parameters of the CDC Growth Charts [22]. The LMS parameters are the median (M), the generalized coefficient of variation (S), and the power in the Box-Cox transformation (L) for skewness. Statistical significance was determined at α = 0.05 for a two sided test. All data were analyzed using SAS v9.1.

An index of relative socioeconomic status (rSES) was developed using principal component analysis, combining the mutual associations among educational levels of parents, household income, unemployment status, public assistance benefits, and resources in the home. The rSES scale was used in the models rather than the individual components.

Descriptive data are presented including frequencies, percentages, means, and standard deviations. Annual growth velocities were calculated relative to Z-scores of BMI, weight, and height. A mean growth velocity of zero indicates the children are growing exactly like the CDC growth reference during that interval. Analysis of variance was used to compare the difference of growth velocities during the three intervals (kindergarten, summer, and first grade). Adjusted growth velocities (Z-score units/year) for BMI Z-scores, weight Z-scores, and height Z-scores were estimated and compared for each interval using generalized linear mixed models adjusted for children's age, gender, intervention condition, rSES and weight status at baseline, and with school as a random effect.

## Results

Table 1 shows descriptive characteristics of children at baseline. The children are approximately equally divided by gender with a mean age of 5.8 years. The children in this sample are taller and heavier than the CDC reference data, as indicated by the mean Z-scores of 0.61 and 0.62, respectively; 14.3% of children were overweight and 15.0% were obese at baseline.

**Table 1:** Descriptive characteristics of children and SES index (rSES) at baseline (n = 440)

Child Characteristics	N	Statistic
Gender		

Female	213	48.4(%)

Male	227	51.9(%)

Weight status		

Normal weight	311	70.7 (%)

Overweight	63	14.3 (%)

Obese	66	15.0 (%)

Age at baseline (mean ± SD, year)		5.8 ± 0.5

Age at round 2 (mean ± SD, year)		6.2 ± 0.5

Age at round 3 (mean ± SD, year)		6.7 ± 0.5

Age at round 4 (mean ± SD, year)		7.3 ± 0.5

BMI (mean ± SD)		16.7 ± 2.89

BMI Z-score (mean ± SD)		0.49 ± 1.17

Weight (mean ± SD, kg)		22.9 ± 5.50

Weight Z-score (mean ± SD)		0.62 ± 1.12

Height (mean ± SD, cm)		116.8 ± 5.57

Height z-score (mean ± SD)		0.61 ± 0.94

rSES score (mean ± SD)		3.0 ± 1.0

**Figure 1:**
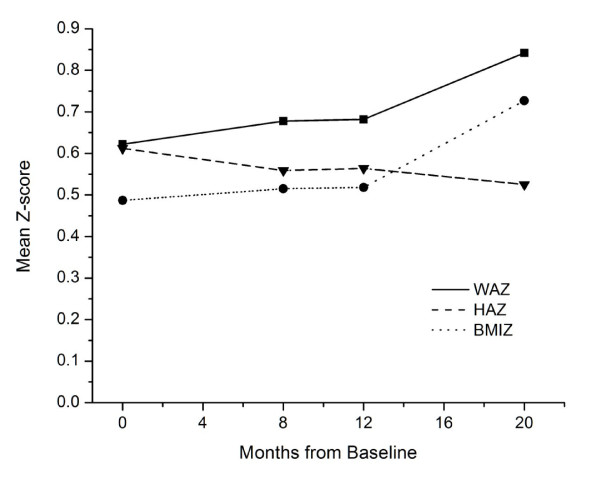
**BMI, weight and height Z-scores time trend overall pattern**.

Figure 1 shows the patterns of mean Z-scores for attained weight, height, and BMI at each measurement occasion, adjusted to exact time intervals. The mean Z-scores for all the measures (weight, height, BMI) are substantially greater than zero at every measurement occasion which is consistent with the overall means seen in Table 1. From the attained values, there is no indication of an appreciable summer effect (measurement between 8 to 12 months.) Rather, marked increases in mean Z-scores for weight and BMI, but not for height, are seen between months 12 and 20, corresponding to the interval of first grade.

### Main effects

Table 2 shows crude and adjusted growth velocities (Δ Z-score/year) for height, weight, and BMI during the intervals corresponding to kindergarten, summer, and first grade. There were significant overall interval differences for crude (*p *= 0.002) and adjusted (*p *= 0.009) weight velocities only; the pattern of means indicate that weight velocity during the summer was less than during the school year. The post-hoc tests show that weight velocity during first grade was significantly greater than during the preceding school and summer intervals (indicated by different superscripts). Children gained slightly more height during the summer than during the school year, but not significantly so. The crude and adjusted velocities for BMI for the total sample were not statistically significant across intervals, and the adjusted mean BMI velocity during the summer tended to be lower than during the school intervals.

**Table 2:** Means of crude and adjusted growth velocities during three intervals

Periods	BMI_Z Δ Z/yr		Weight_Z Δ Z/yr		Height_Z Δ Z/yr	
	
	Mean	SD	Mean	SD	Mean	SD
Crude						

Kindergarten	0.084	0.082	0.111^b^	0.031	-0.072	0.036

Summer	-0.028	0.173	-0.005^b^	0.066	0.014	0.074

First grade	0.296	0.049	0.231^a^	0.031	-0.075	0.026

*F *value	1.98		6.20		1.08	

*p *value	0.138		0.002		0.341	

Adjusted†						

Kindergarten	0.042	0.109	0.084^b^	0.047	-0.079	0.049

Summer	0.009	0.115	0.013^b^	0.048	0.014	0.051

First grade	0.313	0.117	0.239^a^	0.050	-0.058	0.052

*F *value	1.49		4.71		0.71	

*p *value	0.226		0.009		0.491	

**^† ^**Adjusted for age, gender, intervention condition, weight status at baseline, school, and socio-economic status

^a, b ^Items with a different superscript are significantly different from each other

### Differences by weight status

**Figure 2:**
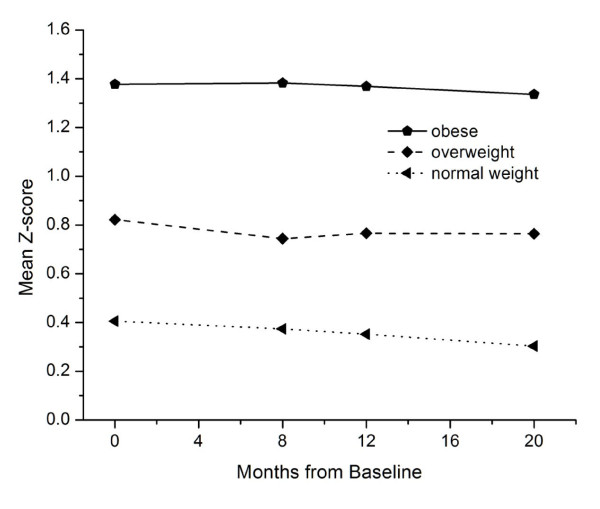
**Height Z-score time trend among different weight status children at baseline**.

We investigated whether the patterns of growth in weight, height, and BMI Z-scores differed by weight status as determined by BMI categories at baseline. The pattern of adjusted mean Z-scores for attained height across the four measurement times were different for each weight status category (normal, overweight, and obese) (Figure 2). Clearly the overweight and obese children are taller than the normal weight children, although the latter children are still considerably taller than the CDC growth charts at the respective ages.

**Figure 3:**
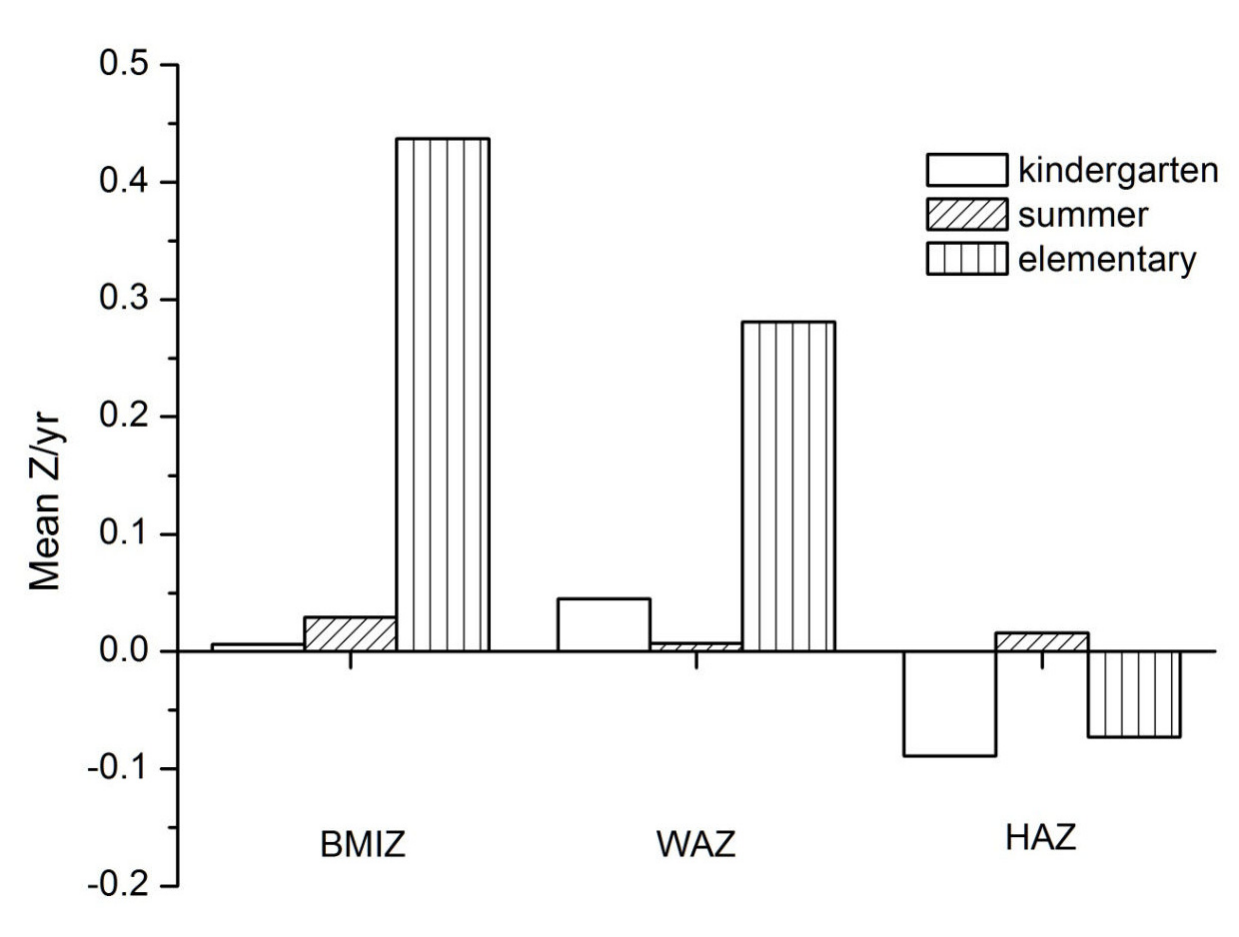
**Mean increments in z-scores for BMI (BMIZ), weight-for-age (WAZ), and height-for-age (HAZ) in normal-weight children during three periods**. BMI weight and height z score increments over three periods among normal weight children at baseline.

There were no statistically significant overall interaction terms in the growth velocity models between school intervals (kindergarten, summer, first grade) and the three categories of weight status, but some interesting patterns still emerged. As an example, in Figure 3 we show the adjusted increments in Z-scores for BMI, weight, and height for normal-weight children relative to the three school intervals, with post-hoc comparisons between means for pairs of intervals. Children with BMI < 85th percentile at baseline showed little difference in growth velocities during the summer compared with the previous school year; however, they had rather dramatic growth in weight and BMI during first grade compared with the previous intervals.

Table 3 presents the adjusted mean increments in BMI velocities (kg/m^2^) gained during the three intervals (kindergarten, summer, and 1st grade) for all weight-status (at baseline) groups. We observe an overall pattern of increasing gain in mean actual BMI, as one progresses from normal-weight to overweight to obese within a school interval, with the exception of obese children in first grade who gained less than their classmates.

**Table 3:** Adjusted^† ^BMI increments (kg/m^2^) during three intervals in weight-status-specific groups

Weight status at baseline	Kindergarten (8.0 mo)		Summer (4.0 mo)		First grade (8.0 mo)	
	
	Mean	SD	Mean	SD	Mean	SD
Normal	0.08	0.12	0.08	0.08	0.66	0.05

Overweight	0.65	0.18	0.22	0.24	0.95	0.31

Obese	1.25	1.09	0.32	1.02	0.57	0.86

Total	0.19	0.05	0.11	0.02	0.71	0.09

**^† ^**Adjusted for age, gender, intervention condition, weight status at baseline, school, and socio-economic status

## Discussion

In the Bright Start study, statistically significant effects were observed in adjusted weight growth velocities among the three intervals: kindergarten, summer, and first grade. Post-hoc contrasts showed that the adjusted weight Z-score velocity during the summer did not differ from that during the previous school year (kindergarten), but it was significantly less than the weight-Z velocity in the subsequent school year (first grade). Because of the concurrent pattern in height-Z velocity, the significant interval-related variation in weight-Z velocity was not accompanied by significant variation in BMI Z-score velocity across intervals when all children were considered. In these data, the American Indian children tended to have lower BMI-Z velocity during the summer months than during the school year.

Rather than using raw increments of BMI, weight, and height in the measured units, we employed annual growth velocities of Z-scores as the chief indicators of growth. This approach accommodates the changing patterns in velocities across the age range, the concurrent differential growth in height and weight that continues over the periods, and it allows direct comparisons for growth in BMI, weight, and height relative to CDC Growth Charts and to other studies.

Smith et al. [17] found no significant change in mean BMI Z-scores over the summer for American Indian children residing on the Wind River Reservation in Wyoming; these children, however, were older (grades 3-8) than the Bright Start children. A large US national sample of kindergarten and first-grade children was studied by Von Hippel and colleagues [18]. In this study, BMI monthly velocity during the summer (0.076 kg/m^2^/mo) was more than three times that of kindergarten (0.020 kg/m^2^/mo) and more than twice that of first grade (0.033 kg/m^2^/mo). While comparing BMI velocities for the intervals does not account for the trend for increasing BMI velocity across the age range as do Z-score velocities, the dramatic difference between summer and first grade for the national sample is interesting. The corresponding estimates for BMI velocities in the Bright Start children are 0.024 kg/m^2^/mo (kindergarten), 0.027 kg/m^2^/mo (summer), and 0.089 kg/m^2^/mo (first grade). The clear differences in the summer and/or out-of-school effects between the two sets of results are difficult to attribute to any specific factors.

General conditions on American Indian reservations are very different in many ways from what might be considered 'average' or representative across the United States, and the most proximal factors related to BMI gain, physical activity, and nutritional intake are clearly obesogenic on reservations [4]. Nevertheless, there is little known about how these factors interact with school and seasonal factors on or off reservations.

Some of the patterns in height, weight, and BMI growth were observed in the Bright Start children are best explained by the expected age-related patterns of physical growth and maturation. The children in our sample are heavier and taller than the CDC growth charts, as demonstrated by their mean Z-scores for height (0.61), weight (0.62) and BMI (0.49). Plains Indians are among the tallest population groups in the world [23]. Moreover, height Z-scores in Bright Start children are positively associated with increasing weight status (see Figure 2). This latter association is not unique and has been reported for other samples of children as early as the third grade [24]. The observation of relatively more rapid growth velocity in height during the summer and weight during the winter months is consistent with that reported in several other studies [25,26], although the exact causes are unknown.

The period between approximately 4 and 7 years of age is characterized by an average decrease in BMI followed by an increase in BMI. This temporary dip in BMI has been termed the "adiposity rebound" [27], and its nadir is characteristically earlier in children with greater BMI or body fat and advanced skeletal maturation. Within populations the earlier the age of the adiposity rebound the more likely are children to become overweight or obese later in childhood and adulthood [28]. The contrast in the pattern of increasing gains in BMI across school-related intervals in normal-weight children compared with decreasing gains in BMI in obese children (Table 3) probably reflects the relative timing of the two groups with regard to the nadir in BMI. The normal children were probably somewhere near the BMI nadir during kindergarten and subsequently accelerated BMI growth, while the obese children have probably past the nadir and were in a phase of decelerating BMI growth. This age and maturation pattern explains the marked increases in weight and BMI Z-scores seen in normal children during the first grade school year (Figure 3).

Our study was methodologically strong. All of the anthropometric data were collected by the same well trained team to minimize any possible confounding between seasonal effects and observer bias. The study was conducted in all schools on the Pine Ridge reservation with greater than 95% child participation, so the results are fully representative of the American Indian children residing there. By having four rounds of data collection we were able to compare growth for two successive school year intervals. Our use of Z-score velocities standardized by age and gender helped demonstrate the continuing growth patterns in height and weight when assessing relative velocity of BMI. Our statistical models were adjusted for varying time intervals between data points and accommodated the design of the Bright Start intervention trial.

Our study also has weaknesses, most of which relate to interpretation of results rather than methodology. We used Z-scores from the CDC 2000 Growth Charts following national recommendations [20]. Nevertheless, the most recent data included in this US reference were collected almost two decades in the past and there were very few American Indians in the samples. We are, therefore, left with the possibility that our estimates of mean Z-scores and even growth patterns in these years of childhood differ from those expected from the growth charts because of secular changes in the population and/or factors related to racial/ethnic differences. Even so, our statistical analyses should yield valid inferences relative to the summer effects *per se*. Also, even with the time lag since the reference data were collected, American Indian children remain taller and heavier than other contemporaneous US children [5,23].

The focus of this study was to determine whether there was evidence of differential gain in BMI during the summer vacation away from school. This is an important question both for school administrators and for those involved in school-based interventions for excessive weight gain and obesity. Our statistical models were adjusted for any intervention effects associated with Bright Start so we could focus on the intended question. Nevertheless, because of the timing of summer vacation, we are unable to separate what might be a "no-school" effect from a seasonal effect. The results of this study indicate no significant summer effects in velocity of BMI Z-score compared to BMI velocities during the preceding and following school years.

BMI is certainly not a perfect measure of fatness in children, although it has been recommended for routine use [20,29]. Some of the positive associations between tallness and obesity in these children may arise from the inability of the squared term for height in the formula used to calculate BMI to capture the actual weight-height relationship across age and sex groups. This potential problem with interpretation of BMI was addressed many years ago by Franklin [30] although it is often overlooked nowadays. To the degree that BMI can be accepted as a reasonable measure of body mass, our conclusions should not be vitiated.

It is difficult to know how generalizable our results are for other school-related circumstances in the US and elsewhere. Clearly, American Indian reservations in the United States are locations for populations at elevated risks for obesity, metabolic, and cardiovascular disease [4]. It is interesting that our results compare closely with those of older American Indian children on the Wind River Reservation [17], but considerably differ from the national sample of children of the same age [18]. While there is little information concerning factors that may be related to differential summer effects, Von Hippel and associates [18] found black children to have greater summer gains in BMI than white or Hispanic children. Whether this is due to differences in prevalences of overweight and obesity or to other factors is unknown. Clearly additional research is required to understand better the nature of summer vacation and its milieu including physical activity, nutritional factors and other individual and environmental characteristics that may be associated with healthy weight and BMI growth when away from school.

## Conclusions

The principal research question addressed was to determine if the velocity of BMI growth during the summer months, when children were not attending school, was significantly different from BMI velocity during periods when school was in session for a sample of American Indian children. After adjusting for age, sex and study design effects, weight velocity was actually smaller during the summer period than during the periods in school. There were no significant summer period effects for growth in BMI and height in these children.

## Competing interests

The authors declare that they have no competing interests.

## Authors' contributions

The authors' responsibilities were as follows-JZ analysed and interpreted the data, and led the writing of the manuscript; JHH helped design and carry out the Bright Start study, assisted in interpreting the results, and critically edited the manuscript; PJH coordinated the analyses; CA critically edited the manuscript; M. Smyth & BHR collected data, administered intervention, and critically edited the manuscript; M. Story conceptualized and designed the Bright Start study, and critically edited the manuscript. All authors read and approved the final manuscript.

## Pre-publication history

The pre-publication history for this paper can be accessed here:

http://www.biomedcentral.com/1471-2458/11/951/prepub

## References

[B1] Wilcox LS Marks JS Centers for Disease Control and Prevention (U.S.) From data to action: CDC's public health surveillance for women, infants, and children 1993 Atlanta, GA.: U.S. Dept. of Health & Human Services, Public Health Service, Centers for Disease Control and Prevention

[B2] Eisenmann JC Katzmarzyk PT Arnall DA Kanuho V Interpreter C Malina RM Growth and overweight of Navajo youth: secular changes from 1955 to 1997 Int J Obes Relat Metab Disord 2000 24 2 211 218 10.1038/sj.ijo.0801116 10702773

[B3] Eichner JE Moore WE Perveen G Kobza CE Abbott KE Stephens AL Overweight and obesity in an ethnically diverse rural school district: the Healthy Kids Project Obesity 2008 16 2 501 504 10.1038/oby.2007.60 18239668

[B4] Story M Evans M Fabsitz R Clay T Holy Rock B Broussard BA The epidemic of obesity in American Indian communities and the need for childhood obesity-prevention programs Am J Clin Nutr 1999 69 suppl 747S 754S 10195597 10.1093/ajcn/69.4.747S

[B5] Zephier E Himes JH Story M Zhou X Increasing prevalences of overweight and obesity in Northern Plains American Indian children Arch Pediatr Adolesc Med 2006 160 1 34 39 10.1001/archpedi.160.1.34 16389208

[B6] Dietz WH Gortmaker SL Preventing obesity in children and adolescents Annu Rev Public Health 2001 22 337 353 10.1146/annurev.publhealth.22.1.337 11274525

[B7] WHO Obesity: preventing and managing the global epidemic. Report of a WHO consultation World Health Organ Tech Rep Ser 2000 894 i xii 1-253 11234459

[B8] Dabelea D Hanson RL Bennett PH Roumain J Knowler WC Pettitt DJ Increasing prevalence of Type II diabetes in American Indian children Diabetologia 1998 41 8 904 910 10.1007/s001250051006 9726592

[B9] Franks PW Hanson RL Knowler WC Sievers ML Bennett PH Looker HC Childhood obesity, other cardiovascular risk factors, and premature death N Engl J Med 2010 362 6 485 493 10.1056/NEJMoa0904130 20147714 PMC2958822

[B10] Perusse L Bouchard C Gene-diet interactions in obesity Am J Clin Nutr 2000 72 5 Suppl 1285S 1290S 11063470 10.1093/ajcn/72.5.1285s

[B11] Danielzik S Czerwinski-Mast M Langnase K Dilba B Muller MJ Parental overweight, socioeconomic status and high birth weight are the major determinants of overweight and obesity in 5-7 y-old children: baseline data of the Kiel Obesity Prevention Study (KOPS) Int J Obes Relat Metab Disord 2004 28 11 1494 1502 10.1038/sj.ijo.0802756 15326465

[B12] Brownell KD Fast food and obesity in children Pediatrics 2004 113 1 Pt 1 132 14702462 10.1542/peds.113.1.132

[B13] Heitzler CD Martin SL Duke J Huhman M Correlates of physical activity in a national sample of children aged 9-13 years Prev Med 2006 42 4 254 260 10.1016/j.ypmed.2006.01.010 16490241

[B14] Jaime PC Lock K Do school based food and nutrition policies improve diet and reduce obesity? Prev Med 2009 48 1 45 53 10.1016/j.ypmed.2008.10.018 19026676

[B15] Story M School-based approaches for preventing and treating obesity Int J Obes Relat Metab Disord 1999 23 Suppl 2 S43 51 10340805 10.1038/sj.ijo.0800859

[B16] Story M Kaphingst KM French S The role of schools in obesity prevention Future Child 2006 16 1 109 142 10.1353/foc.2006.0007 16532661

[B17] Smith DT Bartee RT Dorozynski CM Carr LJ Prevalence of overweight and influence of out-of-school seasonal periods on body mass index among American Indian schoolchildren Prev Chronic Dis 2009 6 1 A20 19080026 PMC2644610

[B18] von Hippel PT Powell B Downey DB Rowland NJ The effect of school on overweight in childhood: gain in body mass index during the school year and during summer vacation Am J Public Health 2007 97 4 696 702 10.2105/AJPH.2005.080754 17329660 PMC1829359

[B19] Carrel AL Clark RR Peterson S Eickhoff J Allen DB School-based fitness changes are lost during the summer vacation Arch Pediatr Adolesc Med 2007 161 6 561 564 10.1001/archpedi.161.6.561 17548760

[B20] Krebs NF Himes JH Jacobson D Nicklas TA Guilday P Styne D Assessment of child and adolescent overweight and obesity Pediatrics 2007 120 Suppl 4 S193 228 18055652 10.1542/peds.2007-2329D

[B21] Lohman TG Roche AF Martorell R Anthropometric Standardization Reference Manual 1988 Champaign, IL: Human Kinetics Books

[B22] CDC 2000 CDC growth charts for the United States 2002 Hyattsville, Md.: Public Health Service, Centers for Disease Control and Prevention, National Center for Health Statistics

[B23] Haas JD Campirano F Interpopulation variation in height among children 7 to 18 years of age Food Nutr Bull 2006 27 4 Suppl Growth Standard S212 223 17361658 10.1177/15648265060274S505

[B24] Stovitz SD Pereira MA Vazquez G Lytle LA Himes JH The interaction of childhood height and childhood BMI in the prediction of young adult BMI Obesity 2008 16 10 2336 2341 10.1038/oby.2008.359 18719630 PMC2747360

[B25] Mirwald RL Bailey DA Seasonal height velocity variation in boys and girls 8-18 years Am J Hum Bio 1997 9 6 709 715 10.1002/(SICI)1520-6300(1997)9:6<709::AID-AJHB4>3.0.CO;2-M 28561389

[B26] Tillmann V Thalange NK Foster PJ Gill MS Price DA Clayton PE The Relationship Between Stature, Growth, and Short-term Changes in Height and Weight in Normal Prepubertal Children Pediatric Res 1998 44 6 882 886 10.1203/00006450-199812000-00010 9853922

[B27] Rolland-Cachera MF Deheeger M Bellisle F Sempe M Guilloud-Bataille M Patois E Adiposity rebound in children: a simple indicator for predicting obesity Am J Clin Nutr 1984 39 1 129 135 6691287 10.1093/ajcn/39.1.129

[B28] Rolland-Cachera MF Deheeger M Maillot M Bellisle F Early adiposity rebound: causes and consequences for obesity in children and adults Int J Obes 2006 30 Suppl 4 S11 17 10.1038/sj.ijo.0803514 17133230

[B29] Himes JH Challenges of accurately measuring and using BMI and other indicators of obesity in children Pediatrics 2009 124 Suppl S3 S22 10.1542/peds.2008-3586D 19720665

[B30] Franklin MF Comparison of weight and height relations in boys from 4 countries Am J Clin Nutr 1999 70 Suppl 157S 162S 10393164

